# Immediate Nonfunctional Loading of Two Single-Maxillary Postextractive Implants: 6-Year Postloading Results of Two Case Reports

**DOI:** 10.1155/2016/6816907

**Published:** 2016-05-16

**Authors:** Vincenzo Ariano, Manuele Mancini, Andrea Cardi, Roberta Condò, Loredana Cerroni, Guido Pasquantonio

**Affiliations:** ^1^Department of Dental Materials, University of Rome “Tor Vergata”, Viale Oxford 81, 00133 Rome, Italy; ^2^Private Practice, Rome, Italy

## Abstract

*Objectives*. The aim of the study was to evaluate and compare crestal bone loss of single-maxillary immediate postextractive implants and immediate nonfunctional loading (INFL) during 72 months of follow-up.* Material and Methods*. Two single titanium implants (Certain Prevail, Biomet 3I, USA) were placed in two patients using INFL technique. Implant stability and crestal bone level were measured on periapical radiographs at 1, 3, and 6 months after surgery.* Results*. All osseointegrated implants were clinically successful after 6 years of functional loading.* Conclusion*. Within the limit of the present case report, the paper supports the concept that INFL of single dental implant can be a successful treatment procedure.

## 1. Introduction

Loss of teeth involves a physiological remodelling of the alveolar bone [1–3] with consequent soft tissues modification. Bone atrophy can be equal to 50% after 5-6 months [4] affecting mainly the horizontal dimension, predominantly on the buccal side, with respect to the vertical dimension [1], continuing slowly but steadily throughout life [5]. Bone reabsorption may lead to a narrower and shorter ridge, thus compromising the functional and aesthetic rehabilitation [6]. Therefore, postextractive hard tissue reabsorption should be avoided. Despite conflicting studies, it could be stated that positioning an implant into a postextractive socket can considerably reduce crestal bone reabsorption [7–9]. Moreover, if the implant is quickly loaded, crestal bone resorption seems to be reduced [10, 11]. Some authors showed that the thickness of the residual bone walls combined with an immediate postextractive implant influences the hard tissue recovery [7–9]. To reduce working time, morbidity, and patient discomfort, many authors stated to immediately insert temporary crowns that can be either loaded or not [12, 13]. The predictability of this technique is guaranteed by the correct treatment plan [14]. This paper shows two case reports of first maxillary premolars replaced by postextractive implants with immediate nonfunctional loading.

## 2. Material and Methods

**Figure 1:**
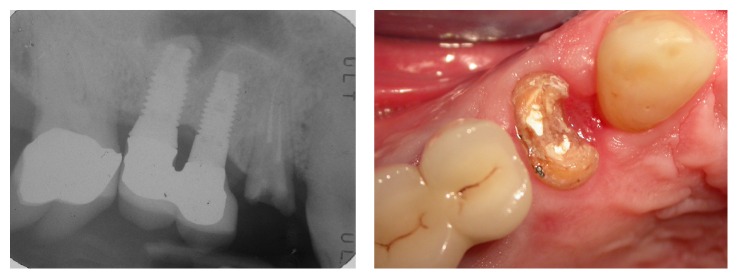
Case 1: clinical and radiographic evaluation before the extraction.

**Figure 2:**
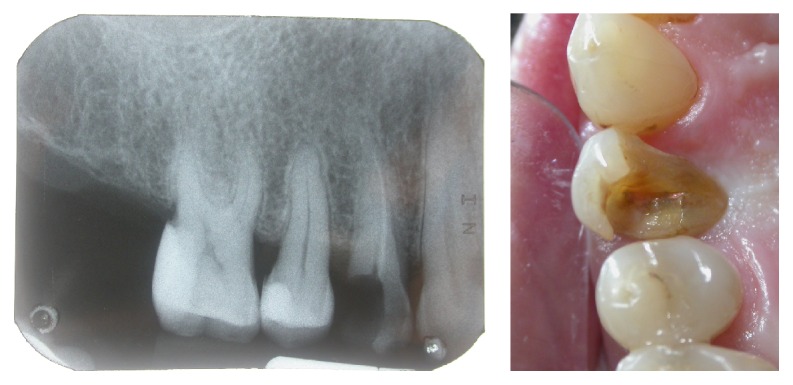
Case 2: clinical and radiographic evaluation before the extraction.

**Figure 3:**
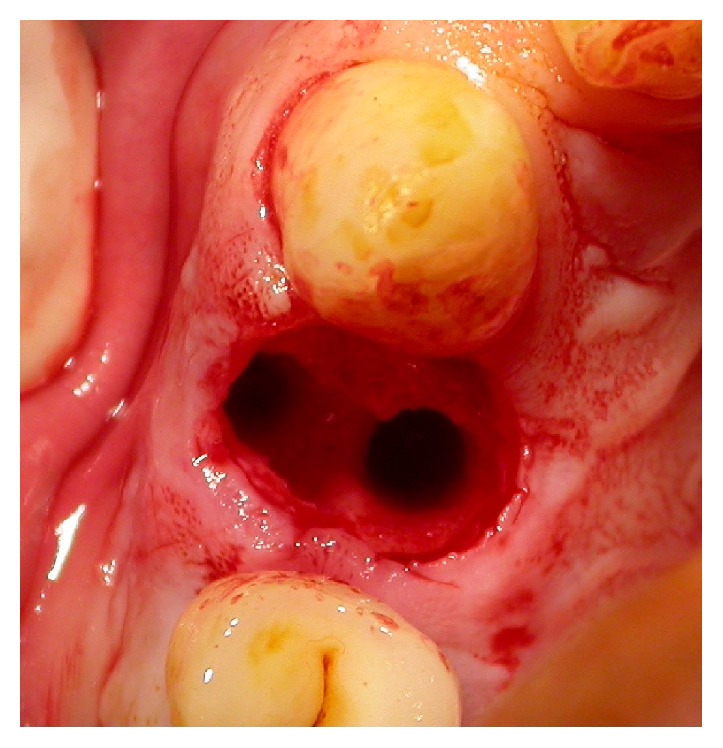
Case 1: extraction with the preservation of the buccal bone.

**Figure 4:**
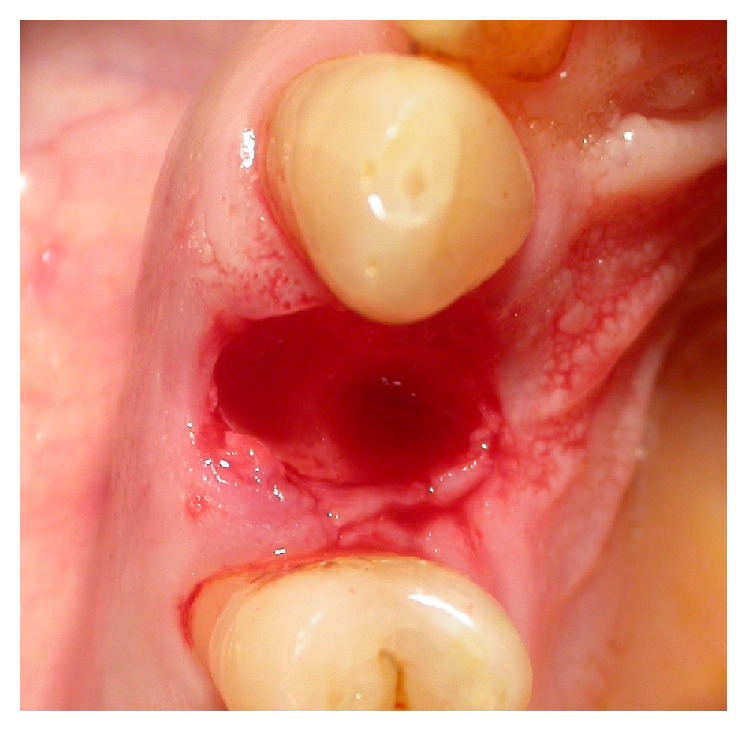
Case 2: extraction with the preservation of the buccal bone.

**Figure 5:**
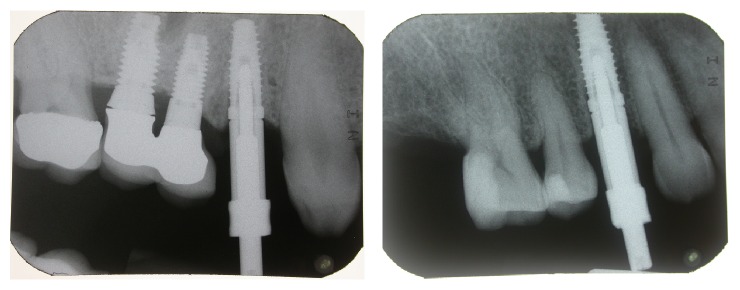
Case 1 and Case 2: after implant placement and connection of the impression transfer periapical radiograph.

**Figure 6:**
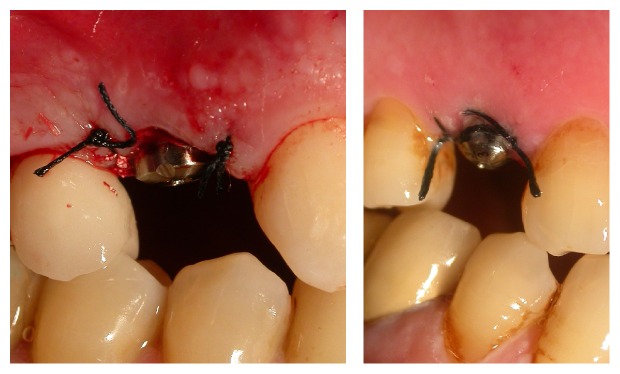
Case 1 and Case 2: placement of healing fixtures and sutures.

**Figure 7:**
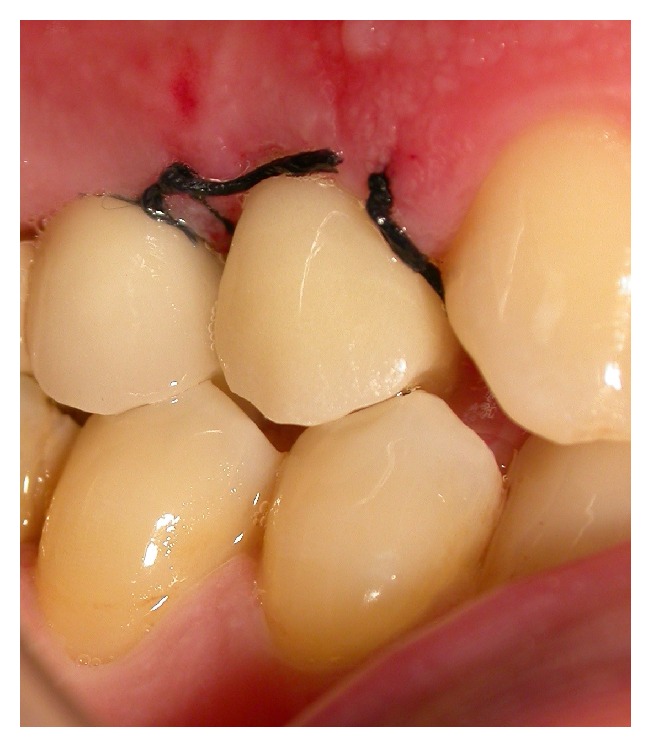
Case 1: connection of the provisional restoration.

**Figure 8:**
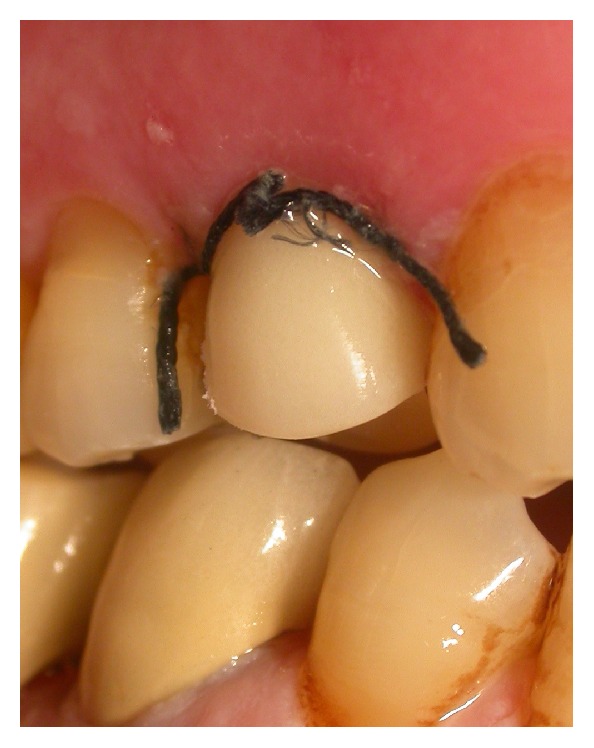
Case 2: connection of the provisional restoration.

**Figure 9:**
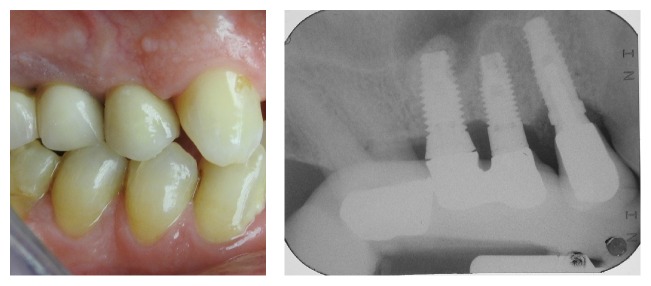
Case 1: definitive prosthetic restoration with periapical radiograph.

**Figure 10:**
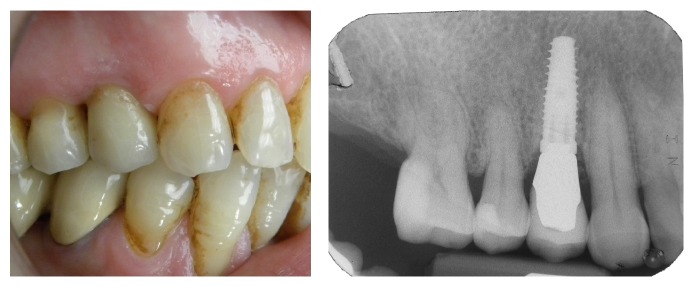
Case 2: definitive prosthetic restoration with periapical radiograph.

**Figure 11:**
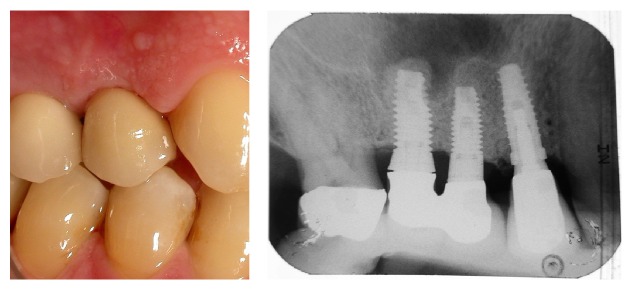
Case 1: 6 years of follow-up and radiographic control.

**Figure 12:**
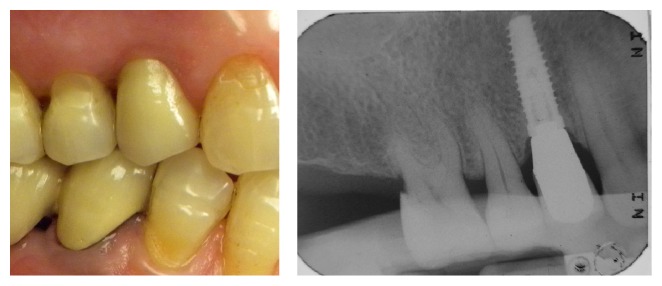
Case 2: 6 years of follow-up and radiographic control.

Two nonsmoking patients were selected: a 59-year-old man (Case 1) and a 77-year-old woman (Case 2), in good physical and psychological conditions. Both patients had tooth 1.4 fractured without acute inflammatory events. After a clinical and radiographic evaluation (Figures 1 and 2), two postextractive implants with immediate nonfunctional loading were planned, due to the aesthetic position of the teeth. Teeth were planned for extraction because the remaining sound tooth and the request of endodontic retreatment and postendodontic build-up were not giving higher percentage of success than the placement of an INFL implant. Gingival biotype was evaluated before the surgery. Soft tissue in both patients was healthy and sound. A preoperative antibiotic and analgesic therapy with amoxicillin 875 mg with clavulanic acid 125 mg (Neoduplamox, Procter & Gamble, USA), 1 tablet every 12 hours for six days starting on the evening before the surgery, and ketoprofen 80 mg (Oki, Dompè Spa, Italy), 2 times a day for 2 days after surgery, was given. Oral disinfection was performed using a 0.2% chlorhexidine mouthwash (Curasept, Curaden Healthcare, Italy) and after surgery a 0.3% chlorhexidine gel (Clexidin Gel Forhans, Uragme, Italy) was added for the 10 days, two times per day. After local anaesthesia with articaine 2% with adrenalin 1 : 100000 (Citocartin, Molteni Dental, Italy) a minimally traumatic flapless extraction of the two elements was performed. After the removal of granulation tissue, an inspection of the alveolar bone to control the presence of any fenestrations was made. Facial bone was intact and it was more than 1 mm thick (Figures 3 and 4). The socket was drilled 3-4 mm over the apical end of the extracted tooth. Moreover the position of fixture was guided by the hollow socket: slightly palatal, 3-4 mm over the apical end of the extracted tooth, and centered between the adjacent teeth. Two fixtures (Certain Prevail, Biomet 3I, USA) previously selected were manually placed to better control their positions (Figures 5 and 6). In Case 1 a 4 × 13 mm fixture and in Case 2 a 4 × 11 mm fixture were used. After connecting the impression transfer (Figure 5), a polyether impression (Impregum Penta, 3M, USA) was taken with a custom tray prepared by the dental laboratory. Subsequently, sites were washed thoroughly with sterile saline and absence of impression material debris was verified. In Case 1, a grafting with hydroxyapatite particles, collagen, and glycosaminoglycans (Biostite, Gaba, Therwil, Switzerland) was placed in the peri-implant space. Healing fixtures were inserted and 4/0 silk sutures (Ethicon, Johnson & Johnson Medical, Rome, Italy) were positioned (Figure 6). After 24 hours the healing fixture was removed; the two resin temporary crowns, built around a PEEK (polyetheretherketone) abutment, were tightened to the fixture without any contact. Screw-retained temporary crowns were chosen to avoid cement that may lead to inflammation (Figures 7 and 8). Patients were instructed to maintain a liquid diet for 3 days and a semisolid diet for about a month avoiding chewing in that side. Both patients reported no pain during the first two weeks and the whole period before the final prosthetic rehabilitation. Sutures were removed after ten days. After four months provisionals were removed. A gold-ceramic crown was luted on a standard titanium abutment in Case 1. In Case 2, a full ceramic crown cemented on a zirconia abutment (Atlantis, Astra Tech Dental System, Mölndal, Sweden) was cemented (Figures 9 and 10). After one year the full crown of Case 2 was replaced by a zirconium ceramic one due to accidental fracture. A sixty-month follow-up was chosen, with clinical evaluation of the soft tissues and radiographic check for any bone resorption in the peri-implant site (Figures 11 and 12). Radiographies were taken with Rinn® (Dentsply-Rinn, York, USA) centering system.

## 3. Results

A 6-year periapical X-ray follow-up showed a good maintenance of crestal bone in Case 1 and a moderate crestal bone resorption (<2 mm) in Case 2. Case 1 shows a good preservation of gingival architecture with a discrete presence of mesial papilla (justified by the presence of tooth 1.3); the distal papilla is underrepresented, for the unfavorable presence of an implant replacing tooth 1.5. In Case 2 mesial and distal papilla is well preserved as good as the gingival margin, showing a slight vertical discharge not affecting aesthetics.

## 4. Discussion

The loss of a tooth is followed by a major alveolar bone reabsorption compromising any future implant-prosthetic rehabilitation. Therefore, bone volume preservation should always be the main goal, even though bone volume augmentation and short and small-diameter implants are nowadays safe and validated alternatives [15–17]. Long-term predictability [9, 18, 19] of immediate-loaded postextractive implants brings advantages to both patients and dentists: reduced postoperative time to finalization, increased patient comfort, reduced morbidity, and good preservation of gingival architecture [20]. Immediate postextractive implants showed osseointegration rates similar to those of delayed implants, with survival rates varying from 90 to 100% [21, 22] with similar histological healing [23]. Many studies on immediate postextractive implants were carried out, but results on physiological crestal bone resorption are controversial [23–27]. Immediate-loading implants seem to promote conservative bone remodelling; in fact, it was shown that implants stimulate bone formation influencing the early stage of the osteointegration. Moreover, they increase mineralized bone at the bone-implant interface [26, 28]. Conversely, some authors showed that immediate-loading postextractive implants do not prevent physiological bone resorption [29, 30]. Those different results can be explained by many factors such as implant positions, implant diameter [31], surface treatment, thickness of residual alveolar crestal bone [7, 8], and type of surgery [32]. Many authors showed the long-term success of immediate-loaded postextractive implants [9, 18, 19]. The scheduling of this rehabilitation technique should be very precise, from patients' selection to surgery [24]. An accurate radiological examination is of paramount importance selecting the fixture: morphology and position of contiguous teeth roots, distance between them, and the remaining bone are essential to reduce micromovements that should be less than 50 microns [10, 31, 33]. Surgery should be very precise [34]. Performing a flapless technique could be useful to further reduce bone resorption [35], even though some authors showed that this technique does not increase benefits [36]. An atraumatic extraction of the selected tooth, respecting both hard and soft tissues, will be crucial [14, 37, 38]. Looking for fenestrations and fractures of the bone socket is important [10] as well as the evaluation of the gingival biotype, its dimension, and the distance between gingival margin and the residual bone crest level. All these data are important to choose the correct drilling depth before positioning the right fixture to obtain good aesthetics of the soft tissue [39]. To assure sufficient tissue tropism distance from contiguous roots should be at least 1.5 mm [37, 40]. The gap between implant and cortical bone does not necessarily need to be filled with bone grafts when it is shorter than 1.5 mm [23, 25, 41, 42]. However, some authors showed that it is better to fill the gap to maintain a greater volume of buccal tissue [43]. In order to reduce gingival dimensional changes and to obtain a long-term success, profiling emergence of the abutment, temporary, and final crowns should be well-finished and should present a concave contour [44, 45]. Contraindications of immediate-loaded postextractive implants are related to all conditions in which primary stability with torque values higher than 35 N/cm cannot be obtained, like insufficient apical bone or the presence of bone gaps [46, 47] and the absence of intact peri-implant bone walls [32, 38, 48]. Benefits of immediate nonfunctional loaded postextractive implant surgery can be listed as good preservation of gingival architecture, reduction of surgical and prosthetic phases with a significant shortening of time, reduced morbidity, reduced costs, improved patient's comfort, and satisfaction [14].

## 5. Conclusion

Within the limits of the present study, the two case reports showed good and satisfactory long-term clinical results using immediate nonfunctional loaded postextractive implants as widely reported by many authors.
